# An Investigation into the Acidity-Induced Insulin Agglomeration: Implications for Drug Delivery and Translation

**DOI:** 10.1021/acsomega.3c02482

**Published:** 2023-07-06

**Authors:** Megren H. A. Fagihi, Chanaka Premathilaka, Tiina O’Neill, Massimiliano Garré, Sourav Bhattacharjee

**Affiliations:** † School of Medicine, University College Dublin, Belfield, Dublin 4, Ireland; ‡ Clinical Laboratory Sciences Department, College of Applied Medical Sciences, Najran University, Najran 55461, Kingdom of Saudi Arabia; § Institute of Veterinary Medicine and Animal Sciences, Estonian University of Life Sciences, Tartu 51006, Estonia; ∥ Conway Institute, University College Dublin, Belfield, Dublin 4, Ireland; ⊥ Super-Resolution Imaging Consortium, Royal College of Surgeons in Ireland University of Medicine and Health Sciences, Dublin D02 YN77, Ireland; # School of Veterinary Medicine, University College Dublin, Belfield, Dublin 4, Ireland

## Abstract

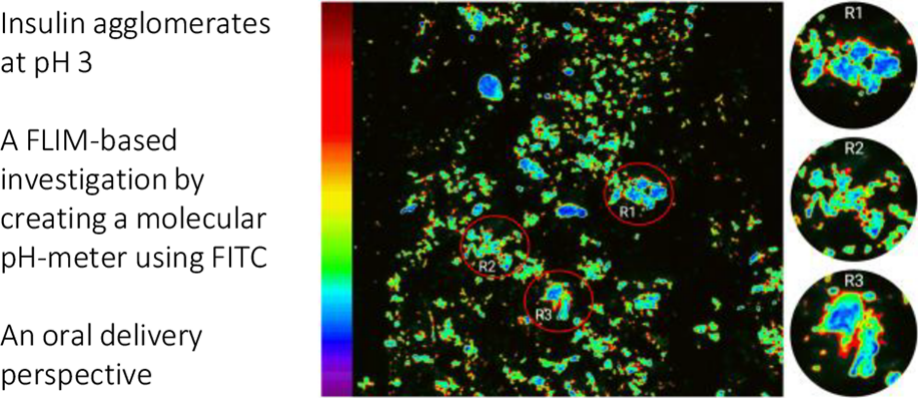

Insulin undergoes agglomeration with (subtle) changes in its biochemical environment, including acidity, application of heat, ionic imbalance, and exposure to hydrophobic surfaces. The therapeutic impact of such unwarranted insulin agglomeration is unclear and needs further evaluation. A systematic investigation was conducted on recombinant human insulin—with or without labeling with fluorescein isothiocyanate—while preparing insulin suspensions (0.125, 0.25, and 0.5 mg/mL) at pH 3. The suspensions were incubated (37 °C) and analyzed at different time points (*t* = 2, 4, 24, 48, and 72 h). Transmission electron microscopy and nanoparticle tracking analysis identified colloidally stable (zeta potential 15 ± 5 mV) spherical agglomerates of unlabeled insulin (100–500 nm). Circular dichroism established the preservation of insulin’s secondary structure rich in α-helices despite exposure to an acidic environment (pH 3) for 72 h. Furthermore, fluorescence lifetime imaging microscopy illustrated an acidic core inside these spherical agglomerates, while the acidity gradually lessened toward the periphery. Some of these smaller agglomerates fused to form larger chunks with discrete zones of acidity. The data indicated a primary nucleation-driven mechanism of acid-induced insulin agglomeration under physiologically relevant conditions.

## 1 Introduction

With a global surge in diabetes cases involving all age groups, including young adults, the importance of insulin as a therapeutically relevant biomolecule has soared.^1^ It is estimated that ∼10% of the global population now suffers from diabetes,^2^ and in alliance with its complications, such as diabetic retinopathy and nephropathy, the disease continues to impart a humongous toll in terms of human suffering, mortality, or morbidity, and healthcare costs. With a reported 4.2 million deaths caused by diabetes in 2019, it has emerged as the seventh most fatal disease.^3^

Insulin is released by the β-cells of the islets of Langerhans nested within the pancreas, which, upon mixing with the bloodstream, reduces the blood sugar levels by a combination of mechanisms, including glycogenesis.^4,5^ It was discovered in 1921 by a team of Canadian researchers: Frederick Banting, Charles Best, and John Macleod and purified later by James Collip. The discovery was subsequently recognized with a Nobel Prize in Physiology or Medicine (1923) to Banting and Macleod, who shared their awards with Best and Collip, respectively. Insulin was the first molecule to be fully sequenced, and the seminal work by Frederick Sanger also received a Nobel Prize in Chemistry (1958).

Insulin is primarily administered via parenteral routes—subcutaneous or intravenous infusions—while endeavors toward developing oral formulations have unfortunately resulted in repeated failures.^6,7^ One of the main reasons behind such a frustrating outcome is the vulnerability of insulin to a harsh biochemical environment that induces degeneration of its peptide backbone.^8,9^ Insulin is known to agglomerate and precipitate when exposed to an acidic gastric environment (pH 2–3). Besides hydrochloric acid secreted by the parietal cells, gastric juice also contains electrolytes and traces of pepsin and lipase. However, an acidic environment remains the key diver behind insulin agglomeration. A similar agglomeration of insulin can be induced in a suspension by administering heat, a fluctuating ionic ambiance, exposure to hydrophobic surfaces and to additives.^10^ Unfortunately, such unwarranted and often unwelcome agglomeration risks deactivating a considerable part of an administered dose. Thus, research toward stabilizing the insulin molecule has intensified over the last few decades, especially keeping its translatory and therapeutic relevance in perspective.

Some research groups have tried to overcome this challenge of acidic gastric juice-induced degeneration by encapsulating the insulin payload within pH-sensitive materials, such as polymers or silica—often in a core–shell nanoconstruct—that protect the cargo of insulin molecules in an acidic gastric interior but gradually dissolves in a higher pH, for example, in the jejunum (pH 6.2) with a release of insulin in vicinity to the Peyer’s patches which due to a lack of mucus coating exhibit facilitated absorption.^11^ Although promising, these nanoformulations need further evaluation and refinement, while their translation from the benchtop to bedside is awaited.

Human insulin (C_257_H_383_N_65_O_77_S_6_) molecule has a molecular weight of 5808 Da, and it is stored within the pancreas as a stable but physiologically inactive hexameric form (35 × 50 Å^2^) held together by two Zn^2+^ cations at the center of this nanocavity.^12,13^ Thus, the molecular weight of such a hexamer is ∼36 kDa. When released into the bloodstream, the hexameric insulin undergoes gradual unpacking into a dimer and, finally, the physiologically active monomer form.^14^ The helical structure of the insulin molecule comprises A (21 amino acids) and B (30 amino acids) polypeptide chains that are held together by two disulfide linkages (A7–B7 and A20–B19).^15^ There is an additional intrachain disulfide linkage within the A chain (A6–A11). The number of antiparallel α-helices in the A and B-chain is two and one, respectively.

The mechanism of acid-induced insulin agglomeration remains unclear. Advanced analytic platforms, viz., atomic force microscopy, cryo-electron microscopy, X-ray crystallography, small-angle X-ray scattering, small-angle neutron scattering, dynamic light scattering, and Thioflavin-T fluorescence, have provided crucial insights into insulin fibrillation.^16^ However, (amyloid) fibrillation is only a subset of the entire landscape of insulin agglomeration, and a large fraction of the agglomerative forms remains unexplored. Even with insulin fibrillation, the molecular mechanism(s) lacks clarity. Some have argued in favor of a primary nucleation-driven mechanism where foci of agglomeration first appear, with further stacking of insulin oligomers resulting in expansion.^17−19^ However, investigations based on Thioflavin-T emission, contrary to sigmoidal kinetics commensurate with a nucleation-driven model,^20^ demonstrated a double-sigmoidal fit supportive of a secondary nucleation thesis.^21^

Previous investigations from our lab on agglomeration of porcine gut mucus—largely constituted of the biopolymer mucin—on a microfluidic chip with the help of fluorescence lifetime imaging microscopy (FLIM), while using fluorescein isothiocyanate (FITC) as a marker dye with a pH-dependent fluorescence lifetime (τ), have demonstrated that highly acidic (pH ≤ 3) localized pockets triggered agglomeration.^22^ Such isolated acidic patches, acting as sanctuaries for a localized condensation of protons (H^+^), with some interpretation, are comparable to a primary nucleation process previously proposed for insulin agglomeration.

We hypothesized that, like mucin, the emergence of acidic foci in an insulin suspension also drives agglomeration. An abundance of H^+^ in acidic solutions provides a conducive environment for forming such acidic foci. If true, this will explain the emergence of a double sigmoidal kinetic pattern due to these initially isolated foci gradually coalescing into larger agglomerates. In line with our previous work, due to its ability to showcase sample matrices with granular details that might be useful in imaging discrete zones of various degrees of acidity within agglomerated insulin, a FLIM-based investigation with FITC as a marker dye acting as a molecular pH-meter was prioritized.

The aims and objectives of the study were (i) to characterize insulin agglomerates by advanced analytical tools, including visualization of its morphology and physico-chemical state(s); (ii) to understand if and how acidity exerts deleterious effects on insulin’s secondary backbone; (iii) to probe the mechanism(s) of acid-induced insulin agglomeration using advanced microscopic platforms; (iv) to interpret the obtained data through the lens of translation and pharmaceutical formulation development; and (v) to optimize the experimental protocols and ensure validity plus reproducibility.

The cumulative data demonstrated the prowess of microscopic techniques with fascinating insights into insulin agglomeration, including the mechanistic purview of the process. The revelations provided a nucleation-driven template for the agglomeration and added a new dimension to insulin delivery platforms that need enhancement as the silent pandemic of diabetes strengthens its foothold in human society. Furthermore, the lessons on insulin agglomeration can deliver an interesting narrative and encourage revisiting similar agglomeration patterns noticed with other therapeutically relevant peptides, proteins, and biomacromolecules.

## 2 Materials and Methods

### 2.1 Chemicals and Reagents

Recombinant human insulin (product number: 91077C) and FITC-labeled recombinant human insulin (product number: I3661; degree of substitution: 1 mole/mole) were obtained commercially from Sigma Aldrich. The lyophilized powdered products were stored in the dark at −20 °C. To dissolve the insulin, a pH 3 solution was prepared. Buffer solutions of comparable pH were avoided due to solubility issues, and a rich ionic environment is known to alter insulin’s molecular conformation.

### 2.2 Preparation of Insulin Suspensions

Human insulin, with or without FITC-labeled, was dissolved in a pH 3 solution at serial dilutions of 0.125, 0.25, and 0.5 mg/mL, and was incubated at 37 °C.

### 2.3 Transmission Electron Microscopy

A 10 μL aliquot of unlabeled insulin suspension (0.125, 0.25, and 0.5 mg/mL) maintained at 37 °C was deposited on a 200 mesh formvar–carbon coated copper electron microscopy grid and left for air drying at various time points: *t* = 2, 4, 24, 48, and 72 h. The grids were then visualized using a Tecnai G2 12 BioTWIN transmission electron microscope.

### 2.4 Nanoparticle Tracking Analysis

The nanoparticle tracking analysis (NTA) was conducted at room temperature (25 °C) using a ZetaView PMX 110 V3.0 instrument (Particle Metrix GmbH, Germany) under a sensitivity of 85, a frame rate of 30/s, and a shutter speed of 70. Roughly 800 μL of unlabeled human insulin suspensions (0.125, 0.25, and 0.5 mg/mL) was injected into the measuring chamber. A prior calibration of the instrument with 100 nm polystyrene nanoparticles (Applied Microspheres B.V., the Netherlands) was performed. The size distribution of insulin agglomerates was determined upon averaging three cycles (11 frames-per-cycle), while the data were analyzed by the proprietary ZetaView NTA software (*n* = 3).^23^

### 2.5 Zeta Potential

The zeta potential (ZP) of unlabeled insulin agglomerates in suspensions (0.125, 0.25, and 0.5 mg/mL) was measured in duplicates (*n* = 2) at room temperature (25 °C) under the following settings: sensitivity of 85, a frame rate of 30/s, and a shutter speed of 70. The ZetaView NTA software was used to analyze the data.

### 2.6 Circular Dichroism

The circular dichroism (CD) measurements of unlabeled insulin suspensions (0.125, 0.25, and 0.5 mg/mL) were conducted in a Jasco J-810 spectropolarimeter fitted with a xenon lamp and a computer-controlled Peltier temperature control device at room temperature (25 °C). The insulin suspensions were taken in a quartz cuvette with 1 mm optical path length, while a spectral region of 200–300 nm (bandwidth of 1 nm) was scanned. The data were averaged out of eight accumulative readings and analyzed with the Jasco Spectra Manager software to determine the molar ellipticity (θ). The data points were plotted using the OriginPro 2015 software (OriginLab Corp., Northampton, MA, USA). Furthermore, the CD data were analyzed by the online tool https://bestsel.elte.hu/index.php for determination of the % of α-helices in secondary structure.^24−26^

### 2.7 Fluorescence Lifetime Imaging Microscopy

The FLIM was conducted in a Leica Stellaris 8 Falcon system fitted with LAS X software (version 4.4.0.24861) and an Okolab incubation system for live cell imaging. The FITC-labeled insulin suspension (0.125 mg/mL) incubated for 24 h at 37 °C was deposited on a shallow chamber created on a glass slide by gluing a coverslip. The acquisition was done with a White Light Laser tuned at 488 nm (40 MHz), both at 37 °C and room temperature (25 °C), under a Leica HC PL APO CS2 100×/1.40 oil immersion objective, and a Leica HyD S detector for collection of photons with a wavelength between 505–605 nm. The pixel size (<100 nm) was decided based on the Nyquist sampling theorem for attaining the highest image resolution. The 3D deconvolution, FLIM phasor plot analyses, and image processing were performed using the Leica LAS X software.

## 3 Results

### 3.1 Transmission Electron Microscopy Investigation

**Figure 1:**
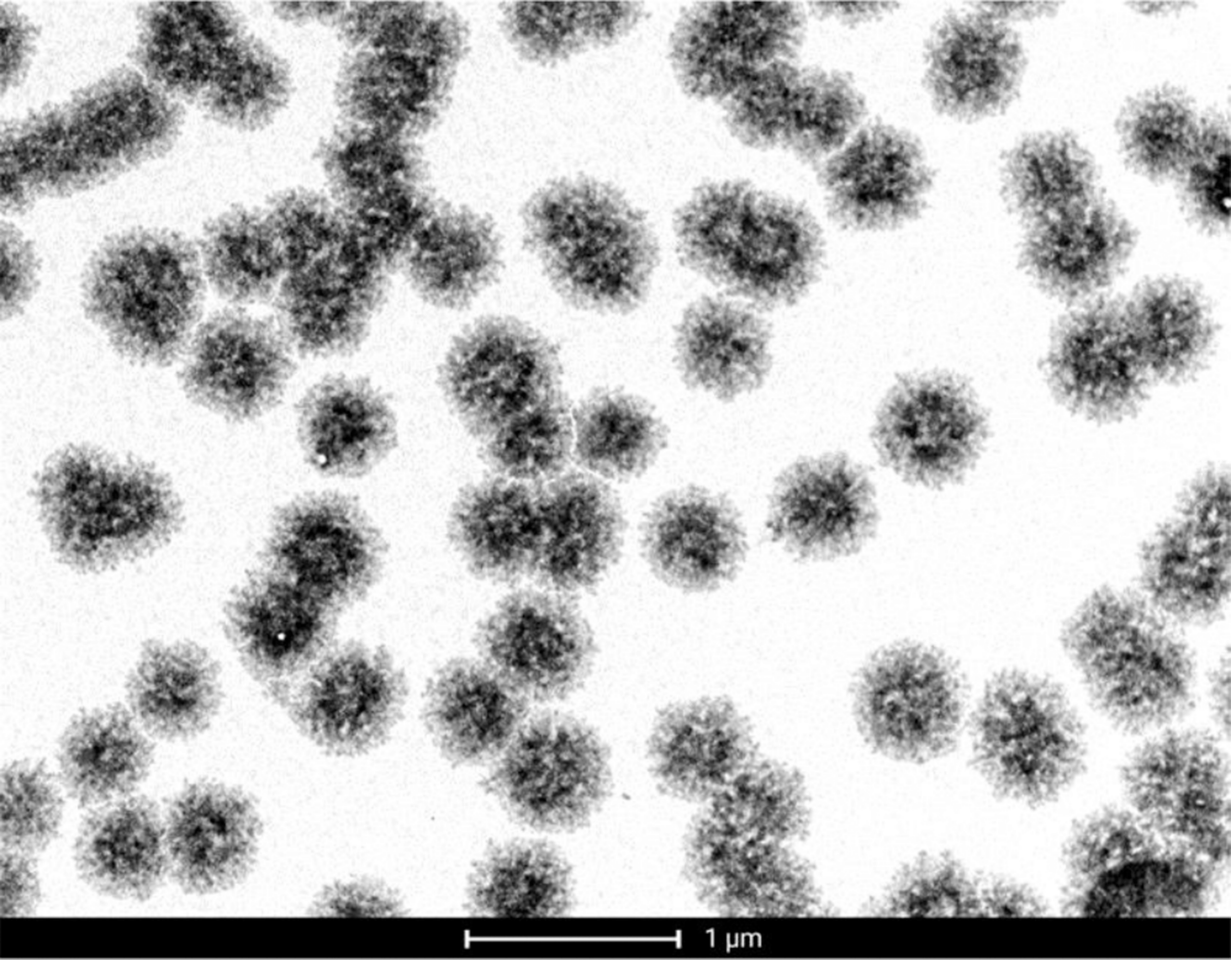
TEM image (16,500×) of unlabeled human insulin suspension (0.125 mg/mL) after 2 h of incubation at 37 °C showing spherical agglomerates (400–500 nm). A 1 μm scale bar is shown.

The transmission electron microscopy (TEM) data confirmed agglomeration of unlabeled human insulin for all the included concentrations (0.125, 0.25, and 0.5 mg/mL) and time points (*t* = 2, 4, 24, 48, and 72 h). The 0.125 mg/mL suspension showed the formation of spherical and overall monodisperse agglomerates of 400–500 nm diameters (Figure 1). Similar agglomerates were also noted for the 0.25 mg/mL insulin suspension (Supporting Information S1), although the images from the 0.5 mg/mL suspensions lacked clarity due to saturation (Supporting Information S2). The subsequent CD findings showed that, unless diluted, 0.25 and 0.5 mg/mL concentrations were incompatible with analytical platforms. Thus, only the 0.125 mg/mL insulin suspension was prioritized for FLIM investigations. Along with the spherical agglomerates, some traces of fibrillation were also noticed (Supporting Information S3).

**Figure 2:**
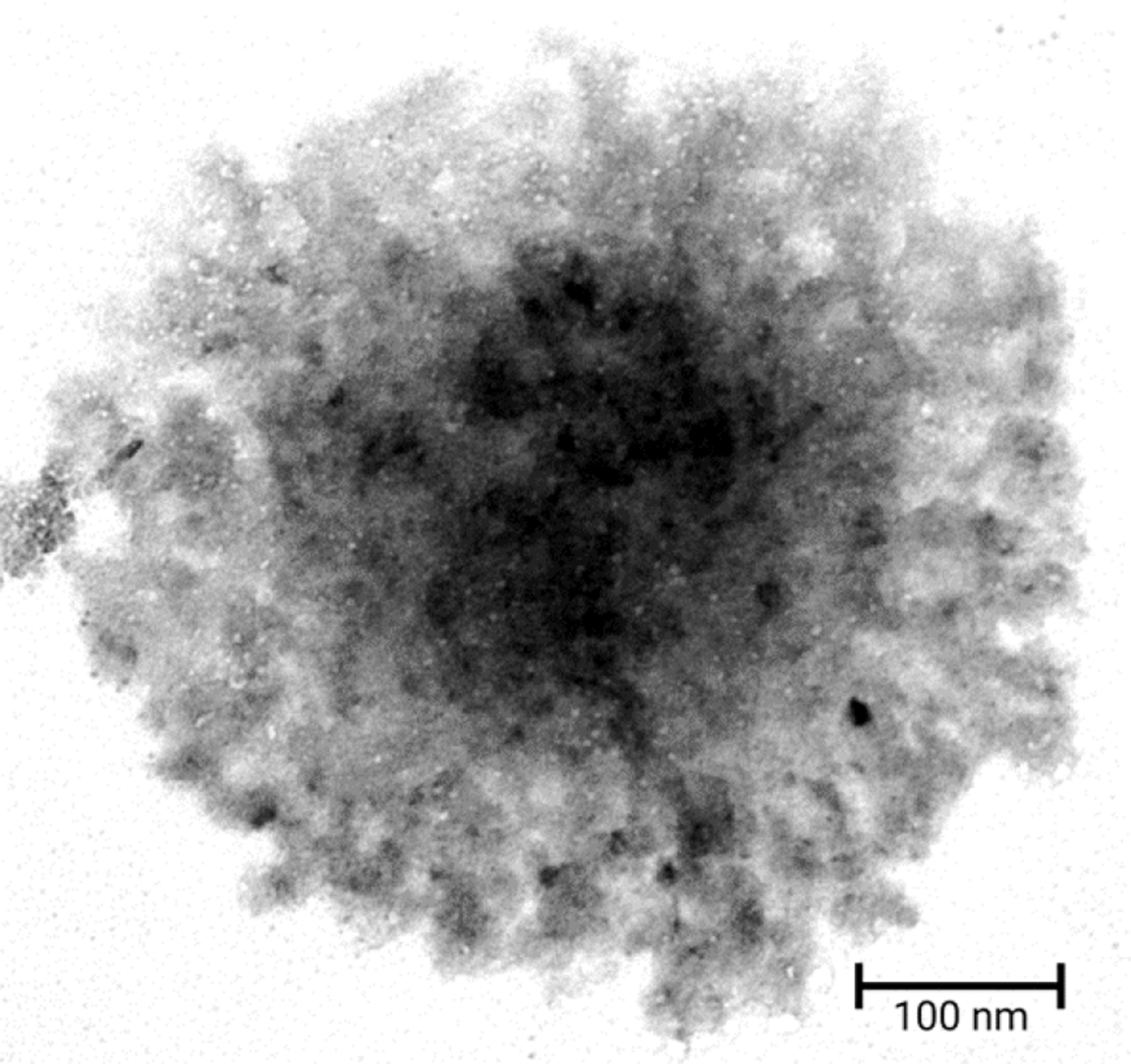
TEM image (105,000×) of human insulin suspension (0.125 mg/mL) after 24 h incubation at 37 °C showing a detailed morphology of an individual spherical agglomerate. The core of the agglomerate seemed dense, while the density kept decreasing toward the periphery. A 100 nm scale bar is shown.

With further zooming into an individual spherical agglomerate, the core appeared denser while the density kept decreasing toward the periphery (Figure 2). Thus, the core of the agglomerates seemed to have stacked relatively more insulin molecules than the periphery, where the composition appeared more untethered. This structural template was maintained for all concentrations (0.125, 0.25, and 0.5 mg/mL) and time points (*t* = 2, 4, 24, 48, and 72 h). However, during TEM, the electron beam traverses higher distances at the center of the spherical agglomerates, which might be a reason behind the cores of these agglomerates appearing to be denser.

### 3.2 Hydrodynamic Size Determination by NTA

The NTA measurements demonstrated the presence of insulin agglomerates with a hydrodynamic size between 100–500 nm (Supporting Information S4 and S5). The concentration of insulin suspensions (0.125, 0.25, and 0.5 mg/mL) did not appear to affect the size of agglomerates for any of the included time points (*t* = 2, 4, 24, 48, and 72 h).

### 3.3 ZP Measurements

**Figure 3:**
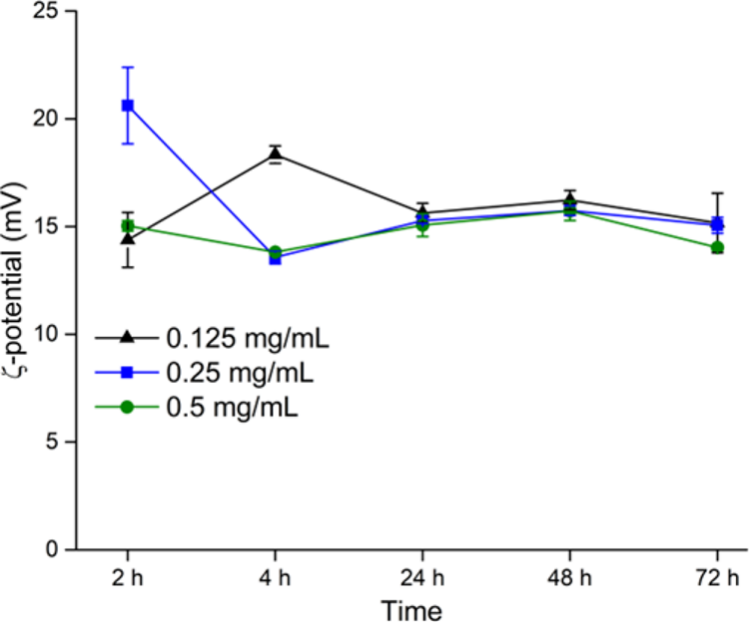
ZP measurements on insulin suspensions (0.125, 0.25, and 0.5 mg/mL) at *t* = 2, 4, 24, 48, and 72 h. Results are shown as mean ± standard deviation (*n* = 2).

The ZP of the insulin agglomerates formed for all the concentrations and time points is provided in Figure 3. The isoelectric point of human insulin varies between 5.2 and 5.4; thus, an acidic milieu of pH 3 resulted in a positive ZP for the agglomerates (Supporting Information S6 and S7). Despite not demonstrating a high ZP, the insulin agglomerates were stable and showed no visible precipitation or clouding over 0–72 h.

### 3.4 CD Measurements

**Figure 4:**
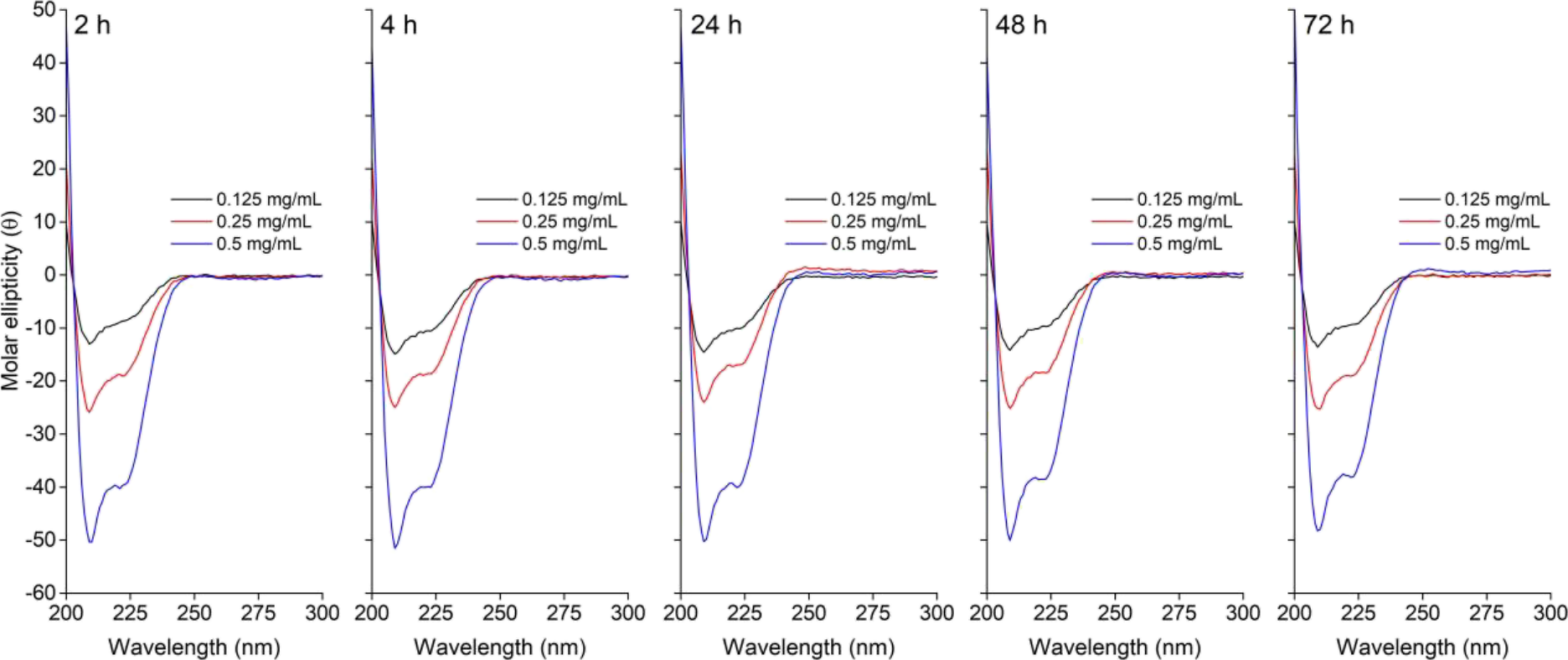
Pooled CD data are shown based on various time points (*t* = 2, 4, 24, 48, and 72 h). The absorbance overall varied according to the dilution factor with characteristic negative stretches around 208 and 222 nm wavelengths due to α-helices. The overall pattern of CD spectra showed resemblance across the different concentrations and time points.

The CD study conducted on the unlabeled human insulin suspensions (0.125, 0.25, and 0.5 mg/mL) demonstrated the secondary structure of insulin at various time points (*t* = 2, 4, 24, 48, and 72 h). The cumulative CD data plotted against the time points (Figure 4) showed an α-helix-rich secondary backbone with two characteristic negative stretches around 210 and 222 nm wavelengths. No band at 216–218 nm, characteristic of a β-sheet secondary structure, was detected. The % of α-helices was noted to be >90% for all the concentrations (0.125, 0.25, and 0.5 mg/mL) and time-points (*t* = 2, 4, 24, 48, and 72 h) without considerable variation.

### 3.5 Fluorescence Lifetime Imaging Microscopy

**Figure 5:**
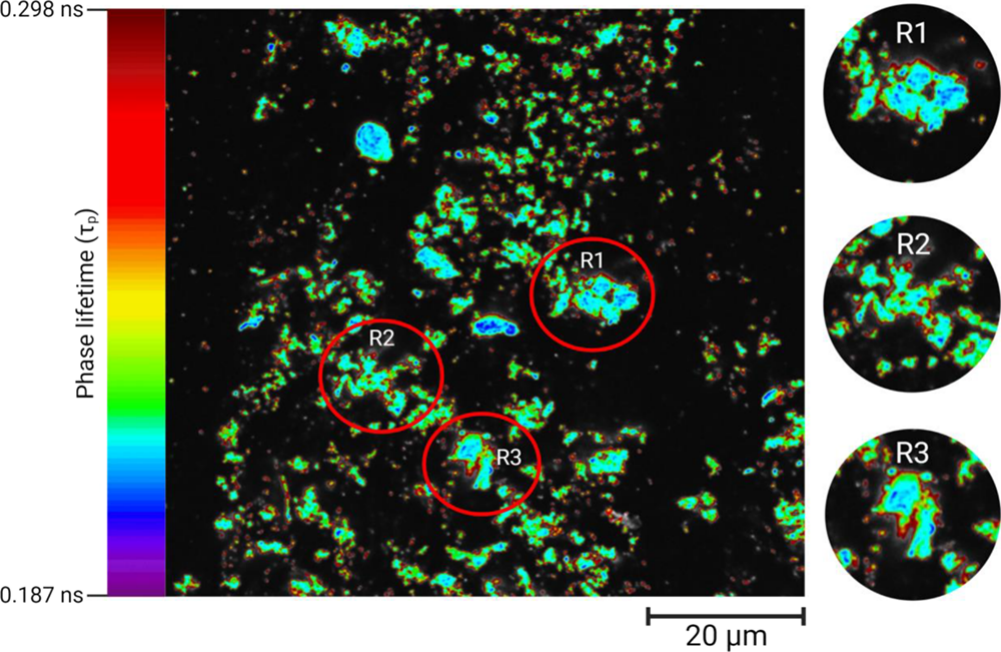
FLIM imaging was conducted at room temperature (25 °C) of a FITC-labeled human insulin suspension (0.125 mg/mL) after 24 h incubation at 37 °C (pixel size 57 nm). The color scale of the phase lifetime (τ_p_) of FITC is shown on the left. Some formations showed a merger of smaller agglomerates. Three larger agglomerates with fused foci are marked within red circles as regions R1, R2, and R3. These regions R1–R3 were then zoomed and shown in a column on the right. The cores of these regions were acidic (blue), while the acidity decreased along a gradient toward the periphery, with transitioning zones marked in green, yellow, and red, indicative of the periphery being the least acidic. A scale bar of 20 μm is embedded within the figure.

A FLIM investigation on FITC-labeled insulin suspension (0.125 mg/mL) upon 24 h incubation at 37 °C revealed the zones of various acidity within the agglomerates. The fluorescence lifetime of FITC is pH-dependent: a lower lifetime signifies an acidic pH and vice versa. Overall, the visual field showed a homogeneous spread of the agglomerates, with some larger than others (Figure 5). The agglomerates had an acidic core (blue) that gradually merged into more basic (green, yellow, and red) zones appearing as layers (regions R1, R2, and R3). A phasor plot is provided in Supporting Information S8.

**Figure 6:**
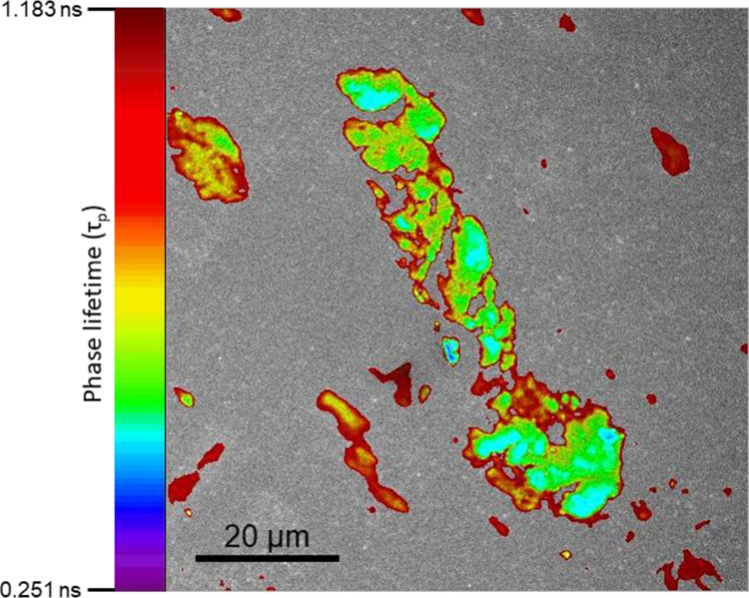
FLIM image (37 °C) of a FITC-labeled human insulin suspension (0.125 mg/mL) after 24 h incubation at 37 °C is shown (pixel size 152 nm). The color scale of the phase lifetime (τ_p_) of FITC is shown on the left. Multiple larger agglomerates with fused smaller foci were noticed. There were distinctive areas of acidity ranging from highly acidic (blue) regions transitioning gradually toward more basic domains (green, yellow, and red) surrounding these isolated pockets of acidity. A scale bar of 20 μm is embedded within the figure.

FLIM investigations on the same sample but now conducted under 37 °C elicited a similar visual, albeit with a higher background with larger agglomerates of FITC-labeled insulin resulting from a fusion of multiple smaller constructs with distinctive zones of varied acidity (Figure 6). The phasor plot is provided in Supporting Information S9.

**Figure 7:**
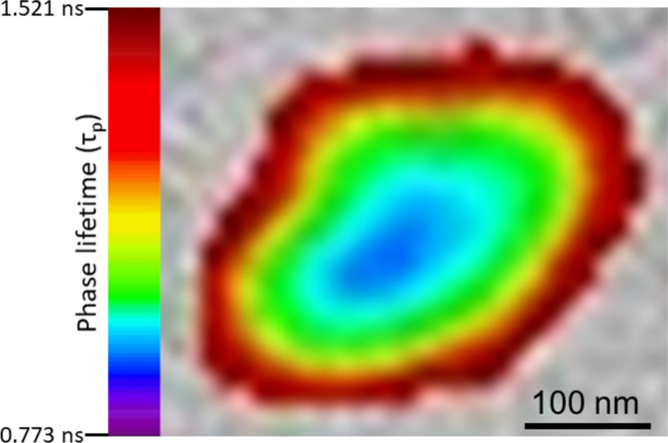
FLIM imaging (37 °C) of an individual insulin agglomerate formed within a FITC-labeled human insulin suspension (0.125 mg/mL) after 24 h incubation at 37 °C (pixel size 38 nm). The color scale of the phase lifetime (τ_p_) of FITC is shown on the left. The core of the agglomerate was highly acidic (blue) that gradually transitioned toward more basic domains (green, yellow, and red) at the fringe zones. A scale bar of 100 nm is embedded within the figure.

The focus was then placed on individual insulin agglomerates to confirm this array of layers with varying acidity—with acidity decreasing from the core toward the periphery—stacked along a gradient. The FLIM data confirmed such layers of decreasing acidity, with the core being the most acidic segment of the agglomerate (Figure 7). As determined by light microscopy, the overall size of these agglomerative constructs of FITC-labeled human insulin (∼500 nm) corroborated with the TEM data.

**Figure 8:**
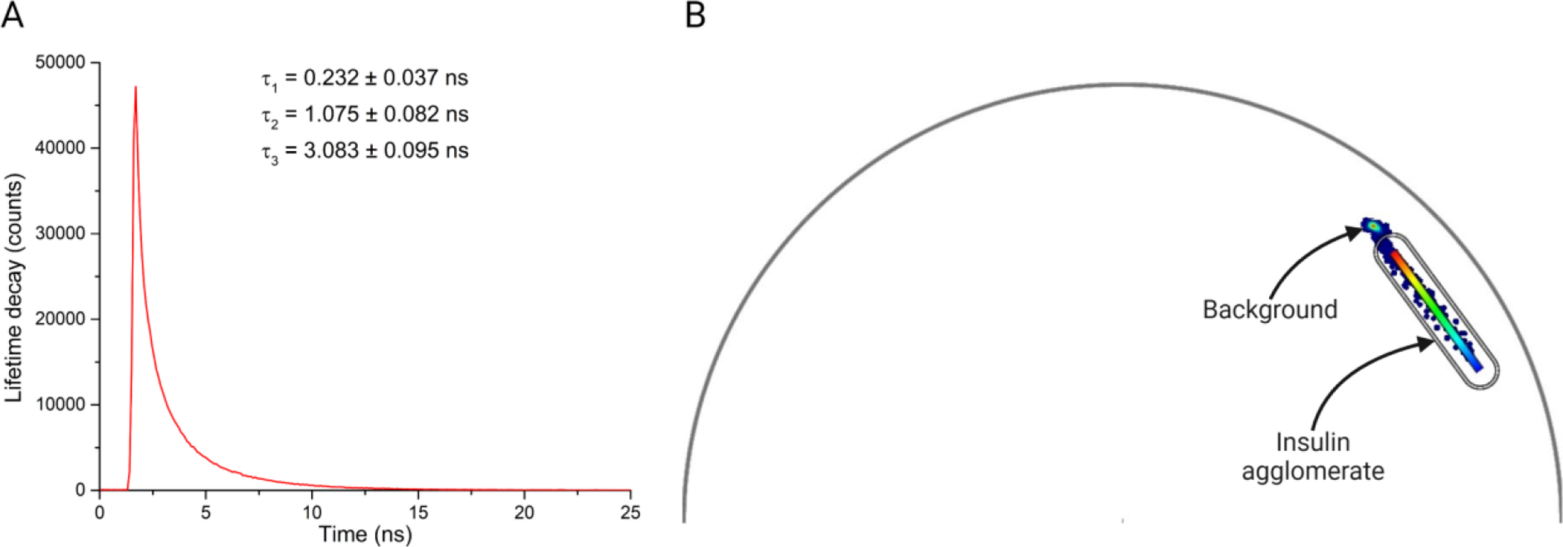
(A) Fluorescence lifetime decay of a FITC-labeled insulin agglomerate imaged by FLIM elicited a three-component fit. (B) Phasor plot showing the distribution of photons detected from the sample. Two distinct clouds of photons were noticed from the background and sample. Both areas lay within the universal circle, indicating a multiexponential decay. The color scale of phasor fluorescence lifetime was created on the photons received from the agglomerates.

The lifetime decay of an individual FITC-labeled agglomerate shown in Figure 7 elicited three distinctive exponential decay fits (Figure 8A) with the following lifetimes: τ_1_ = 0.232 ± 0.037 ns, τ_2_ = 1.075 ± 0.082 ns, and τ_3_ = 3.083 ± 0.095 ns. The phasor plot showed two distinct clouds of photons collected from the sample (Figure 8B) lying within the universal circle due to multiexponential decay.

## 4 Discussion

Insulin agglomeration—with or without fibrillation—has remained a challenge in developing oral formulations or administering them in resource-poor and tropical areas where preservation facilities are often inadequate. Unfortunately, insulin is prone to agglomeration even with subtle fluctuations within its physico-chemical ambiance, including heating, acidity, and ionic imbalance.^27−29^ It remains largely unknown to what extent such agglomeration deactivates insulin.

Regrettably, there are no quick ways to quantify or predict the bioactivity of an (agglomerated) insulin preparation. In vitro assays were used to ascertain such bioactivity,^30−34^ although these assays need further refinement, acceptable reproducibility, set standard operating protocols, and a better interpretation. Fluorophore-labeled insulin, similar to the FITC-labeled insulin used here, could expand the scope of these in vitro assays. This study has demonstrated the stability and compatibility of FITC-labeled insulin with advanced microscopic techniques. Its further in vitro use might provide crucial information on how insulin formulations—with varying degrees of agglomeration—interact with cells, including their uptake kinetics and intracellular compartmentalization.

Drawing a quick conclusion that agglomeration immediately deactivates insulin might be precarious. Insulin as a molecule is known to exist as monomers, dimers, tetramers, and hexamers, offering varying degrees of bioactivity, with the hexamer being the least effective (or bioactive). Thus, although not entirely comparable to agglomeration, insulin is known to preserve its bioactivity despite multiple molecules clustering together. The CD data have demonstrated a remarkable resilience of insulin in preserving its secondary backbone enriched with α-helices at pH 3 for up to 72 h. Such molecular stability of insulin may be attributed to weak polar and π–π electronic interactions from aromatic side chains.^35^ Available studies reporting a disruption of insulin’s native secondary structure under acidic conditions or heat application have often used much harsher, perhaps a bit far-fetched, experimental conditions, for example, a pH of 1.6 and a temperature of 65 °C.^36^ Many proteins would demonstrate unfolding or misfolding under such denaturing conditions, and data derived from such premises risk lacking physiological or translational relevance.

Our data have established an appearance of spherical agglomerates within insulin suspensions of pH 3, which were colloidally stable although polydisperse. These results must be evaluated on a thermoenergetic backdrop and integrated with the FLIM data. It would be worth noting here that recombinant human insulin was used in this study. The agglomerative patterns might vary with a wide range of short- and long-acting insulin formulations now available, with varying degrees of substitution and purity. Thus, some flexibility should be exercised while interpreting the data on the physical characterization of insulin agglomerates. There are reports demonstrating the fibrillation potential of insulin,^37^ although evidence of such fibrillation in this study was meager. Still, the presence of insulin fibrils cannot be entirely negated as these fibrils are ≤1 nm in width and need a different set of tools, such as high-resolution cryo-electron microscopy, to visualize.

A striking observation of the spherical agglomerates from the TEM investigation was their dense cores that gradually merged into lesser dense layers toward the periphery. On the other hand, FLIM data revealed that the cores of these agglomerates were highly acidic, whereas the acidity kept receding with distance. From the FLIM data, it becomes obvious that acidity, or relative abundance of protons, started forming the cores, and layers of insulin molecules kept stacking based on how many free protons were available. The NTA data provided further testimony toward colloidal stability of these insulin agglomerates as the overall agglomerate size distribution and ZP did not vary much over 0–72 h.

This information is relevant, as there is an unsettled debate over the mechanism(s) of insulin agglomeration. While some have argued favoring a primary nucleation-driven mechanism, others were less than convinced. The FLIM data reported here have established a primary nucleation-driven agglomeration—at least under these circumstances—where the secondary backbone of insulin remained largely unaffected. Furthermore, it has been shown that protons acted as seeds for agglomeration. To the best of our knowledge, we are the first group to report a systematic FLIM-based investigation toward understanding insulin agglomeration and the major biochemical drivers that dictate the process.

We preferred to use the phasor plot approach in FLIM data representation, where each image pixel is plotted in a radial plot depending on its average τ. It creates a direct relationship between the pixels in the image and the dots in the plot. A phasor plot is a visual tool representing all the original data obtained from FLIM in a vector space. In other words, every pixel in a FLIM image is converted into a point in the phasor plot by calculating the sine and cosine of the raw decay data. This process enabled us to achieve such high-resolution images.

The exact mechanism behind a proton-triggered derivation of minuscule foci of insulin agglomeration remains obscure and provides a compelling ground for a thorough follow-up. On a preliminary assessment, it is possible that excess protons in an acidic suspension provide a fertile ground for binding to the aminated basic sites of insulin and create the primordial template or seed for the agglomeration to begin. Further addition of insulin to these emerging focal matrices continues, although such layering also results in gradual masking of the cationic charge that, similar to a negative feedback loop mechanism, discourages further layering once shifted away from the core to a point where the stacking finally reaches its thermoenergetic barrier that, as shown by the FLIM data, marks the (relatively) basic periphery of an insulin agglomerate.

The obtained FLIM data on larger insulin agglomerates also furnished evidence toward a further merger of these initially isolated islands of agglomeration where an acidic pocket—the imprint of an acidic core within insulin agglomerates formed later—could be detected. The different zones within a larger agglomerate marked with varied lifetime decays of FITC make a clear case for such fusion. This is interesting as such a fusion of smaller foci of insulin agglomerates can explain the double-sigmoidal kinetics reported earlier. It is worth mentioning that the phasor plot analyses on the acquired FLIM dataset provided a facile tool for visualizing the different components with varied fluorescence lifetimes in a sample.^38,39^ A phasor analysis approach also helped eliminate the background noise and provided ample flexibility toward data visualization and filtering the dataset to extract information only from the region of interest, which, in this case, were the insulin agglomerates.^40,41^

The study demonstrated the power of FLIM as a microscopic technique and the fascinating data it can deliver while investigating therapeutically relevant molecules. Out of its many strengths as an imaging tool, what emerged to be particularly relevant from the perspective of this study was the insensitivity of the fluorescence lifetime of a fluorophore toward its concentration, emission intensity, and bleaching effect.^42−45^ As a dye, FITC was a stable pH-sensitive marker for microscopy. The fluorescence lifetime of FITC—or other fluorescein derivatives per se, such as C-SNARF-4 or Oregon Green 488—decreases with pH.^46−50^ For example, the lifetime of FITC is 1.6 ns at pH 3, 4.5 ns at pH 5.8, and 7.8 ns at pH 9. However, FITC is often incompatible with super-resolution microscopic techniques, such as stimulated emission depletion microscopy, which might be one of its limitations in further studies.

## 5 Conclusions

Through an integrated approach of electron and light microscopic analyses in conjunction with light scattering tools, such as NTA, acid-induced (pH 3) agglomeration of human insulin suspensions (0.125, 0.25, and 0.5 mg/mL) at 37 °C was probed at different time points (*t* = 2, 4, 24, 48, and 72 h). Stable insulin agglomerates of 100–500 nm sizes were formed. Protons within the biochemical ambiance initiated the nucleation process with an acidic core that acted as a seed for further stacking of insulin layers. Thus, spherical insulin agglomerates with nuclei of high acidity gradually transitioning toward (relatively) basic margins were formed. Some of the agglomerates merged to form larger chunks with embedded regions of varied acidity. In summary, the data showcased the phenomenon of acid-induced insulin agglomeration, portraying its segments of differing acidity as a mechanism of such primary nucleation-driven agglomeration. Such mechanistic investigations provide further insights toward designing oral formulations of insulin with strategies to withstand the acidic exterior inside stomach. Further investigations probing how such acid-induced agglomeration of insulin affects its bioactivity are currently being pursued in our labs.

## Supporting Information Available

TEM images of 0.25 and 0.5 mg/mL insulin suspensions after 2 h incubation at 37 °C, TEM image of 0.125 mg/mL insulin suspension after 24 h incubation at 37 °C, and NTA datasheet from instrument showing the hydrodynamic diameter and zeta potential of insulin agglomerates in 0.5 mg/mL suspension after 2 h incubation at 37 °C, and phasor plot analyses (PDF)
NTA of 0.5 mg/mL insulin suspension after 2 h incubation at 37 °C measuring the hydrodynamic diameter and zeta potential of the insulin agglomerates (AVI)
ZP of 0.5 mg/mL insulin suspension after 2 h incubation at 37 °C measuring the hydrodynamic diameter and zeta potential of the insulin agglomerates (AVI)

## Supplementary Material

ao3c02482_si_001.pdf

ao3c02482_si_002.avi

ao3c02482_si_003.avi
