# Supplementary material for: An Investigation into the Acidity-Induced Insulin Agglomeration: Implications for Drug Delivery and Translation
Source: ACS Omega. 2023 Jul 6;8(28):25279–87. doi: 10.1021/acsomega.3c02482 (PMC10357556; doi:10.1021/acsomega.3c02482)

SUPPORTING INFORMATION

An investigation into the acidity-induced insulin agglomeration: Implications for drug delivery and translation

Megren H. A. Fagihi [1,2], Chanaka Premathilaka [3],
Tiina O´Neill [4], Massimiliano Garré [5], and Sourav Bhattacharjee [6,*]

[1] School of Medicine, University College Dublin, Belfield, Dublin, Ireland

[2] Clinical Laboratory Sciences Department, College of Applied Medical Sciences, Najran University, Najran 55461, Kingdom of Saudi Arabia

[3] Institute of Veterinary Medicine and Animal Sciences, Estonian University of Life Sciences, Tartu (51006), Estonia

[4] Conway Institute, University College Dublin, Belfield, Dublin 4, Ireland

[5] Super-Resolution Imaging Consortium, Royal College of Surgeons in Ireland University of Medicine and Health Sciences, Dublin, Ireland

[6] School of Veterinary Medicine, University College Dublin, Belfield, Dublin, Ireland

*Corresponding author

Email: sourav.bhattacharjee@ucd.ie

Tel.: +353 1 716 6271

Figure S1. TEM image (16,500×) of unlabeled human insulin suspension (0.25 mg/mL) after 2 h incubation at 37°C. A 1 μm scale bar is shown. The brightness and contrast of the image were both enhanced by 20% for better image clarity.

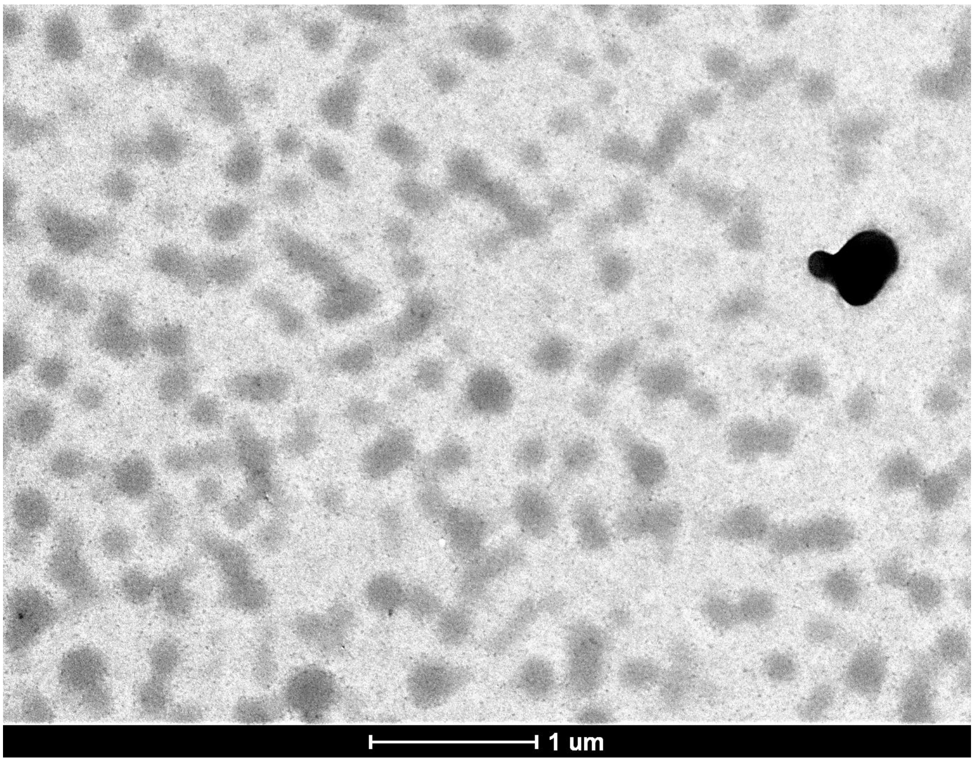

Figure S2. TEM image (16,500×) of unlabeled human insulin suspension (0.5 mg/mL) after 2 h incubation at 37°C showed field saturation. A 1 μm scale bar is shown. The brightness and contrast of the image were both enhanced by 20% for better image clarity.

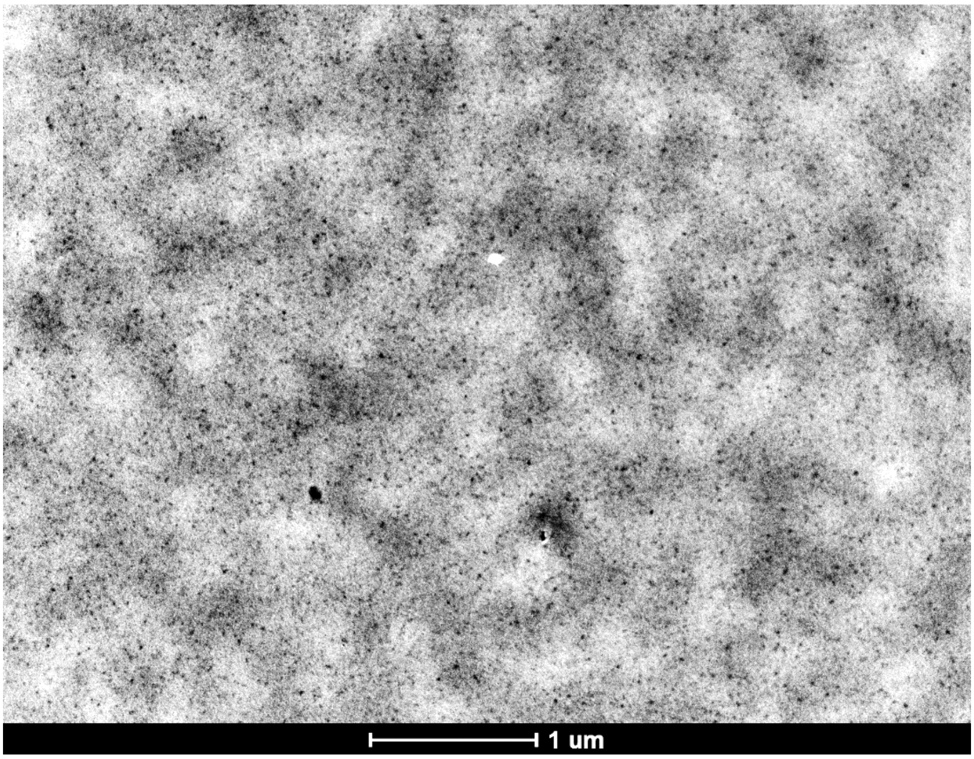

Figure S3: TEM image (1,05,000×) of human insulin suspension (0.125 mg/mL) after 24 h incubation at 37°C showing traces of fibrillar structures marked within red boxes and circles. A 100 nm scale bar is shown. The brightness of the image was enhanced by 20% for better image clarity.

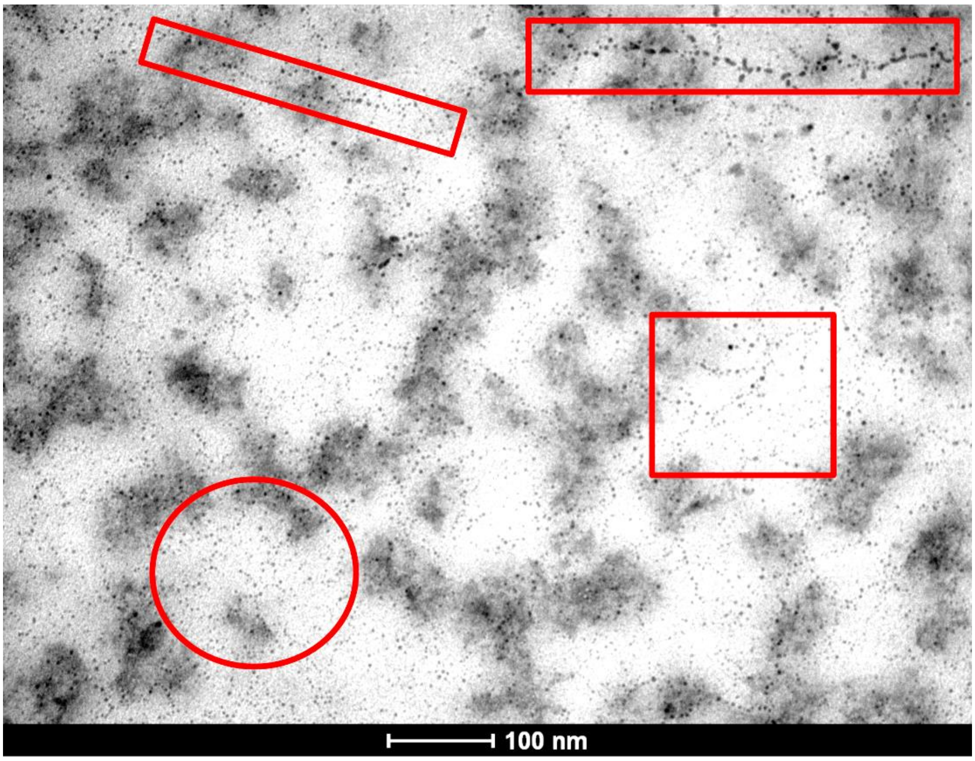

Example report sheet from the NTA instrument while measuring the hydrodynamic size of the insulin agglomerates (0.5 mg/mL after 2 h)

Figure S4

Electrophoresis & Brownian Motion
Video Analysis
Laser Scattering Microscopy

Operator (Report): pcadmin
Video Operator: pcadmin

Sample Parameters
Sample Name: Insulin_05_2hrs
Comment: Sample Remarks0:
Sample Remarks1:
Sample Remarks2:
Electrolyte: H2O
Temperature: 22,97 °C sensed
[redacted]
Conductivity: 130,84 µS/cm sensed

Instrument Parameters
Laser Wavelength: 488 nm
Filter Wavelength: Scatter

Measurement Parameters
Cell S/N: CA0099-0197b

Measurement Mode: Size Distribution 3 Cycles
11 Positions, 1 Removed for Analysis

Result (sizes in nm)

	Number	Concentration	Volume
Median (X50)	113,4	113,4	192,0
Span	53,5	53,4	115,0

Concentration: 1,2E+7 Particles / mL
Dilution Factor: 2
Original Concentration: 2,4E+7 Particles / mL

Quality
Average Counted Particles per Frame: 30
Number of Traced Particles: 519

Analysis Parameters
Max Area: 1000, Min Area: 5, Min Brightness: 25

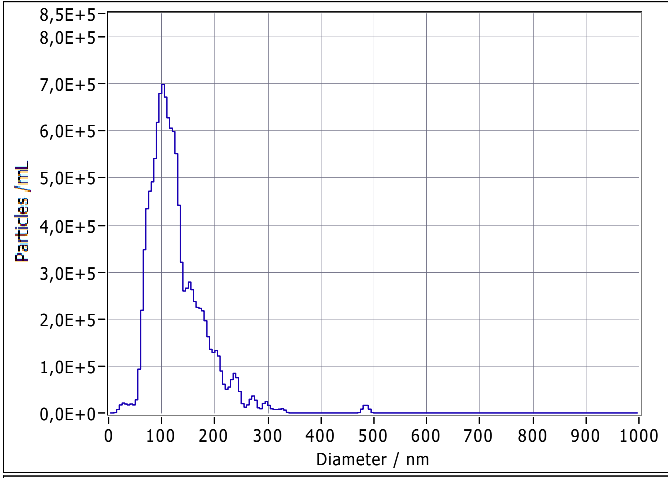

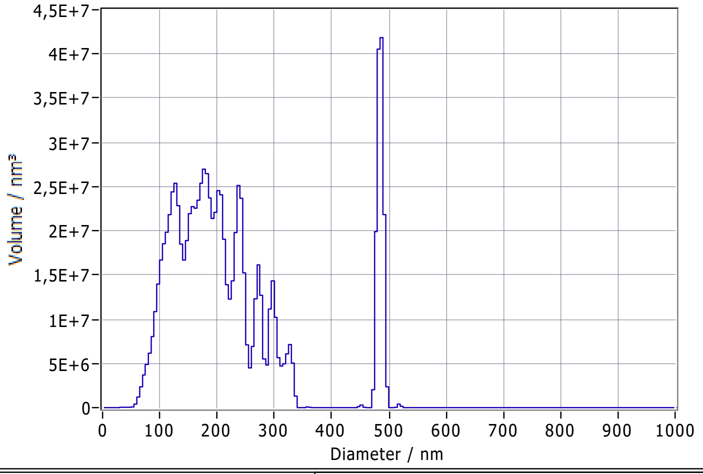

Peak Analysis (Concentration)

Diameter / nm	Particles/mL	FWHM / nm	Percentage
103,6	6,9E+5	68,7	94,7
235,7	8,0E+4	20,6	5,3

X Values (all sizes are given in nm)

	Number	Concentration	Volume
X10	72,7	72,7	109,2
X50	113,4	113,4	192,0
X90	191,9	191,9	479,6
Span	1,1	1,1	1,9
Mean	127,6	127,6	225,7
StdDev	53,5	53,4	115,0

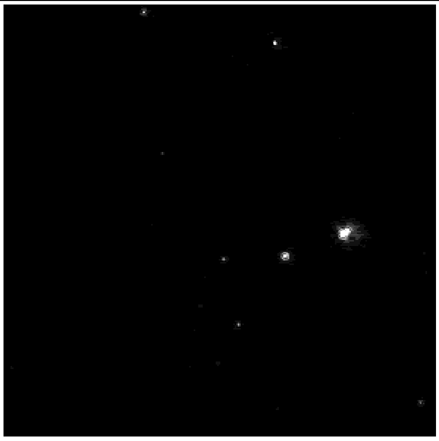

Comment

(Signature)

Analyzed Video: F:\Chanaka\Sourav\2022,12,03\20221203_0006_Insulin_05_2hrs_size_488_001.avi

ZetaVIEW S/N 274, Software ZetaView (version 8.05.12 SP2), Camera 0,703 mum/px

Experiment: 2022-12-03 13:31, Report: 2022-12-03 13:33

Example report sheet from the NTA instrument while measuring the ZP of the insulin agglomerates (0.5 mg/mL after 2 h)

Figure S6

Electrophoresis & Brownian Motion Video Analysis Laser Scattering Microscopy

Operator (Report): pcadmin
Video Operator: pcadmin

Sample Parameters
Sample Name: Insulin_05_2hrs_2
Comment: Sample Remarks0:
Sample Remarks1:
Sample Remarks2:
Electrolyte: H_2O
Temperature: 24,25 °C sensed
[redacted]
Conductivity: 102,13 µS/cm sensed

Instrument Parameters
Laser Wavelength: 488 nm
Filter Wavelength: Scatter

Measurement Parameters
Cell S/N: CA0099-0197b
Sensed Electric Field: 3,14 V/cm (pulsed)
Measurement Mode: Stationary 5 Cycles

Result
Mobility: 1,23 FWHM 0,54 mum/sec/V/cm, @ 25 degC: 1,25 mum/sec/V/cm
ZP Factor: 13,0 (Smoluchowski)
Zeta Potential @ 25 degC: 16,00 FWHM 15,20 mV
Concentration: 2,1E+7 Particles / mL
Dilution Factor: 1
Original Concentration: 2,1E+7 Particles / mL

Quality
Average Counted Particles per Frame: 51
Number of Traced Particles: 568
ΔSL: 2,33 mV

Analysis Parameters
Max Area: 1000, Min Area: 5, Min Brightness: 25

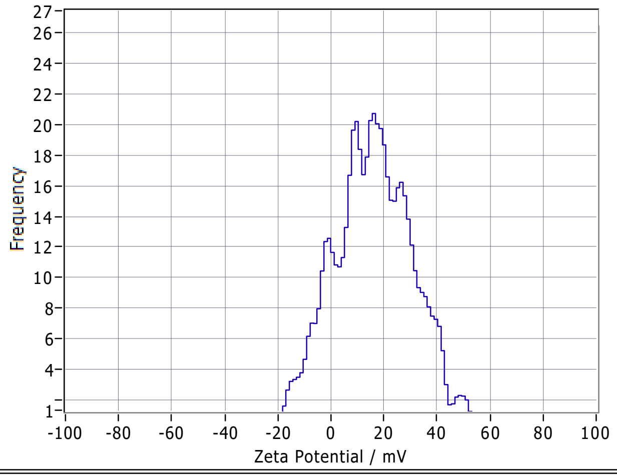

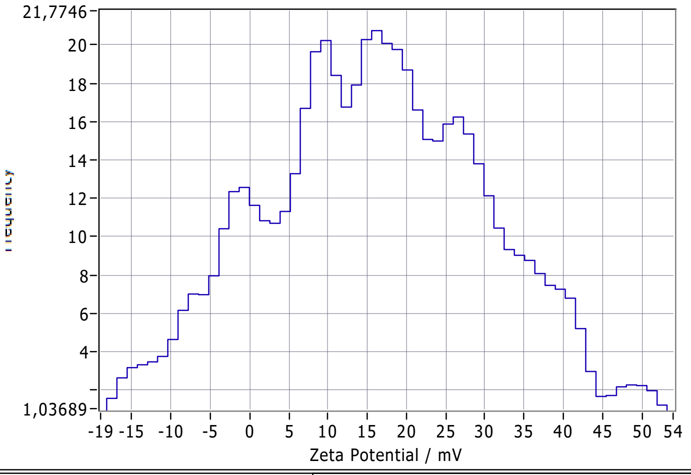

Stationary Layers	Rel. Position
SL1	0,149
SL2	0,851

Peak Analysis (Concentration)

Zeta Potential / mV	Frequency	FWHM / mV	Percentage
16,7	20,5	7,0	36,0
26,2	16,1	10,7	29,4
-0,6	12,6	8,9	17,7
9,7	20,2	6,5	14,5
49,0	2,3	5,3	2,5

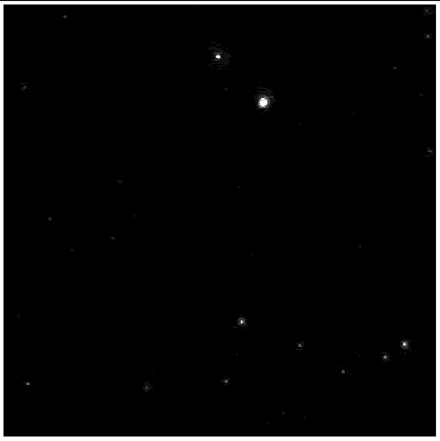

Comment

(Signature)

Analyzed Video: F:\Chanaka\Sourav\2022,12,03\ZP\20221203_0035_Insulin_05_2hrs_2_SL_488_001.avi

ZetaVIEW S/N 274, Software ZetaView (version 8.05.12 SP2), Camera 0,703 mum/px
Experiment: 2022-12-03 15:32, Report: 2022-12-03 15:35

Figure S8. A phasor plot showing the distribution of photons detected from the field shown as Figure 5 in the manuscript. Two distinct clouds of photons were noticed from the background and sample. Both areas lay within the universal circle, indicating a multiexponential decay. The color scale of phasor fluorescence lifetime was created on the photons received from the human insulin agglomerates.

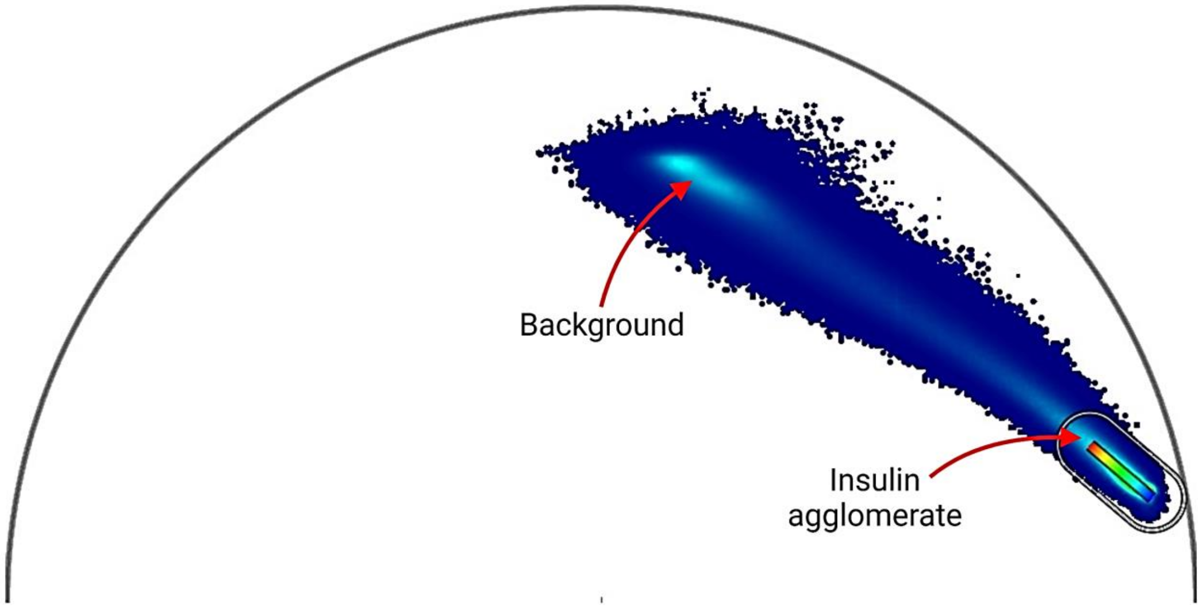

Figure S9. A phasor plot showing the distribution of photons detected from the larger human insulin agglomerate shown as Figure 6 in the manuscript. Two distinct clouds of photons were noticed from the background and sample. Both areas demonstrated a multiexponential decay. The color scale of phasor fluorescence lifetime was created on the photons received from the insulin agglomerates.

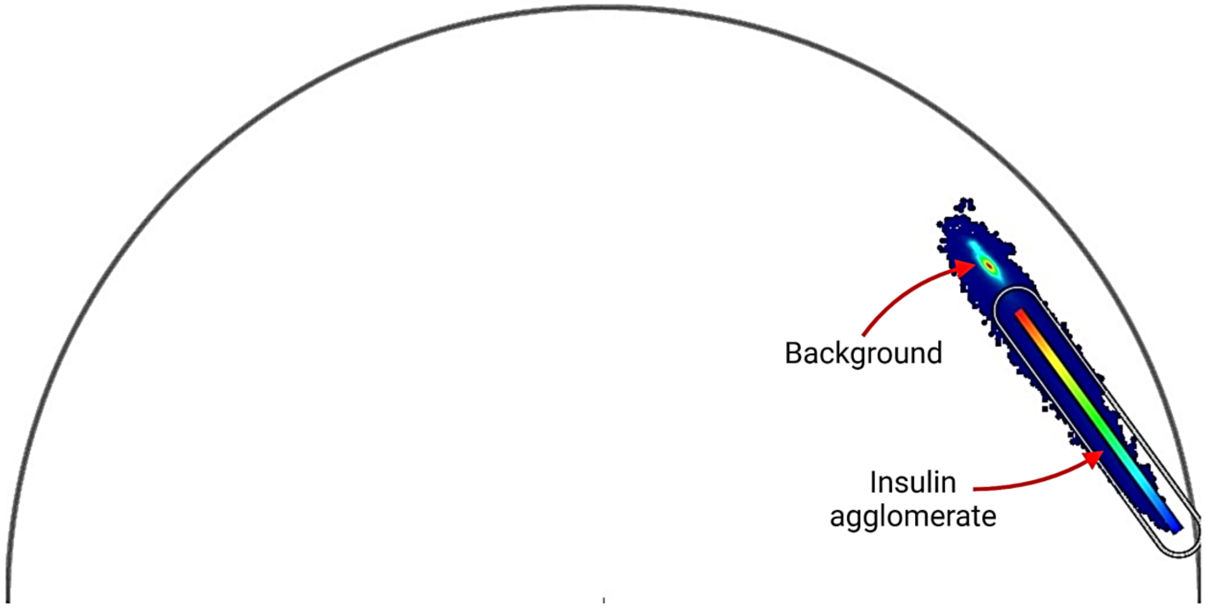

Supplement: Supplementary file 1 — ao3c02482_si_001.pdf [file ao3c02482_si_001.pdf]
